# Complete Genome Sequence of the WHO International Standard for HIV-1 RNA Determined by Deep Sequencing

**DOI:** 10.1128/genomeA.01254-13

**Published:** 2014-02-06

**Authors:** Astrid Gall, Clare Morris, Paul Kellam, Neil Berry

**Affiliations:** a Wellcome Trust Sanger Institute, Wellcome Trust Genome Campus, Hinxton, Cambridge, United Kingdom; b Division of Virology, National Institute for Biological Standards and Control, South Mimms, Potters Bar, United Kingdom; c Research Department of Infection, Division of Infection and Immunity, University College London, London, United Kingdom

## Abstract

The World Health Organization (WHO) International Standard for HIV-1 RNA nucleic acid assays was characterized by complete genome deep sequencing analysis. The entire coding sequence and flanking long terminal repeats (LTRs), including minority species, were assigned subtype B. This information will aid the design, development, and evaluation of HIV-1 RNA amplification assays.

## GENOME ANNOUNCEMENT

Ensuring the safety of blood and blood products from the introduction of human immunodeficiency virus 1 (HIV-1) and the monitoring of HIV-1 RNA concentrations in blood and tissue components of HIV-1-infected patients has been strengthened by the ongoing development of genome amplification techniques in conjunction with the availability of an internationally recognized standard for HIV-1 RNA (1). However, the genetic composition of the biological materials used to derive the World Health Organization (WHO) International Standard for HIV-1 RNA has not hitherto been fully elucidated.

Here, we report the complete genome sequence of the WHO International Standard for HIV-1 RNA. The virus was originally recovered in the United Kingdom in 1994 during postmortem analysis of an HIV-1-infected patient by coculture on human peripheral blood mononuclear cells (2). Viral RNA was extracted using a QIAamp Viral RNA Minikit (Qiagen). The HIV-1 genome was reverse transcribed and amplified in four overlapping amplicons using a “pan”-HIV-1 primer set (3). Amplicons were pooled in equimolar amounts for Illumina library preparation, including a unique bar code for the sample, and sequenced using MiSeq 250-bp paired-end technology in a pool of 25 libraries (4). *De novo* assembly was performed using SPAdes version 2.4.0 (5). Resulting contiguous sequences were aligned with the sequence of the HIV-1 reference strain HxB2 (NC_001802), and a consensus sequence was generated using abacas version 1.3.1 and MUMmer version 3.2 (6). Subsequently, reads were mapped against the consensus sequence using SMALT version 0.5.0 (http://www.sanger.ac.uk/resources/software/smalt/) to analyze read depth and minority species.

The sequence of the WHO International Standard for HIV-1 RNA described here is 8,926 nucleotides (nt) long and has a G+C content of 41.1%. It contains the complete coding sequence (8,606 nt) with nine open reading frames (*gag*, *pol*, *vif*, *vpr*, *tat*, *rev*, *vpu*, *env*, and *nef*), as well as the complete U5 and partial R region of the 5′-long terminal repeat (5′-LTR), and a partial U3 region of the 3′-LTR of the HIV-1 RNA genome. BLAST analysis (7) revealed the highest similarity with the subtype B HIV-1 isolate 5084-83 clone pbf1 from United States (AY835754) (total score 14,652, 95% identity, and 99% coverage). The subtype B of the WHO International Standard for HIV-1 RNA was confirmed by neighbor-joining phylogenetic analysis using PAUP* version 4.0b10b (8) and analysis with the Recombinant Identification Program (9). The mean read depth was 9,167-fold (±6,197 standard deviation [SD]) with a minimum of 25-fold. There are 224 positions with a minority nucleotide that differs from the consensus sequence and has a frequency of >1% and a Phred quality score of >30%, i.e., a base call accuracy of 99.9%.

This is the first report for the complete genome sequence of the WHO International Standard for HIV-1 RNA. The standard is widely used in the development and evaluation of genome amplification assays for HIV-1 RNA quantification, which provide important clinical data for the management of HIV-1-infected patients, primarily in viral load determination. The complete genome sequence reported here will minimize ambiguity or bias in oligonucleotide fidelity and selection to further aid assay development and ensure secure clinical management of HIV-1-infected individuals.

### Nucleotide sequence accession number.

The complete genome sequence of the WHO International Standard for HIV-1 RNA reported here has been deposited in GenBank under the accession number KJ019215.
